# Microbes ‘R’ us

**DOI:** 10.1111/1462-2920.16264

**Published:** 2022-11-20

**Authors:** Josh D. Neufeld

**Affiliations:** ^1^ Department of Biology University of Waterloo Waterloo Ontario Canada

‘Once we understand how similar we are, and how deeply the ties between animals and microbes extend, our view of the world will become immeasurably enriched’ (Yong, 2016).

Lynn Margulis had one of her grant proposals rejected with a reviewer's comment, ‘Your research is crap. Don't ever bother to apply again’ (Anon, n.d.). As an evolutionary biologist, Margulis was a leading proponent of the hypothesis that eukaryotic organelles, such as mitochondria and chloroplasts, arose from microorganisms engulfing other microorganisms (Sagan, 1967). Despite a long history of resistance to such unconventional thinking, an endosymbiotic origin for microbial eukaryotes, plants, and animals is now widely accepted. Especially given that bacteria and archaea monopolized life on Earth for the first billion years or so, such an endosymbiotic origin for eukaryotic life is arguably the most exceptional evolutionary milestone since the last universal common ancestor first evolved. Although microbiologists may take it for granted that modern‐day research related to endosymbiosis can shed light on the origins of eukaryotic cells (Williams et al., 2013; McInerney et al., 2014; Zaremba‐Niedzwiedzka et al., 2017; Imachi et al., 2020), and provide a more comprehensive view of life's phylogenetic diversity (Hug et al., 2016), implications for this research extend far beyond the pages of scientific journals and, I predict, will increasingly transform the way we think of ourselves. As gatekeepers for this knowledge, microbiologists will begin to make increasingly important contributions to the philosophical understanding of who we are as human beings.

From an outsider's perspective, an endosymbiosis‐inspired message from microbiologists may sound bizarre or fantastical: every one of our cells is a microorganism, and each of these is effectively comprised of microbes living within other microbes. As such, we are multicellular microorganisms, in the same general category as mushrooms, each made up of individual microbial cells. By extension, everything that we might consider to be distinctly human, separate from microbes, is an illusion. The human experience is, at its very basis, enabled by a compilation of microbial processes that have evolved in an unbroken chain of cell division over billions of years. Microbial processes laid the foundation for, and continue to sustain, our conciousness, thoughts, memories, interests, feelings, behaviours, relationships, religions, and knowledge systems. Everything human is fundamentally microbial, without exception. As multicellular microorganisms, we also produce waste that feeds more microbes while we carry countless additional microbes in our guts and on our bodies (i.e., we are also ‘holobionts’). Indeed, as hotbeds of microbial life, we feed and breed microbes with everything we do. This message is reflected in the words of Dorion Sagan and Lynn Margulis: ‘Today we realize that in comparison to the richness and antiquity of microbes, we dwellers of the larger realm must be considered so recent and few as to be a mere epiphenomenon‐just one of many curious results of billions of years of bacterial symbioses and evolution. Indeed, strange as it may seem, the reader is just a microbe's means of making another microbe’ (Sagan & Margulis, 1993). Although humans are a remarkable outcome of microbiology and evolution, we remain a compilation of microbes at the very core of our evolutionary history and current functioning. As a result, the study of microbiology may be considered a foundationalist approach to the study of humans, and all life by extension.

Hearing that we are multicellular microorganisms, many may think that this threatens traditional perspectives of the human experience, and its uniqueness, reducing us (and other animals) to mundane microbial machinations. However, I prefer to think that this perspective better reflects the human experience as the improbable and exciting outcome of billions of years of microbial evolution. Research over the last few decades has brought us to an unprecedented place where we can describe and discuss human origins in the context of microbiology, aided by the sequencing of our own genome and those of other microorganisms (e.g., Hug et al., 2016). We, as collections of microorganisms in human form, are the first species to recognize and describe ourselves for what we are: microorganisms assembled and evolved in a way that appears, from our understandably human‐centric perspective, to be a remarkable outcome. But then how does our collective genome, contained within each of our cells, code for this unprecedented and marvellous capacity to know ourselves in this way? What exactly is it to be human? Perhaps roughly similar to ask: what is it to be microbe? There are so many unanswered questions, and some are much better left to philosophers: ‘Understanding of human nature and human interaction ‐ all benefit from and are subtly or sometimes grossly transformed in the light of microbiology. Microbiological insights thus enhance philosophical reflection in a general sense, by making us think more about the very nature of life itself’ (O'Malley, 2014).

Throughout the 21st century, I predict that perspectives of humans as being fundamentally multicellular microorganisms will serve as the foundation for other biological sciences and a touchstone for philosophical reflection. Given that microbiological research continues to add new pages to the earliest chapters of our collective genealogy, microbiologists have a central role to play as stewards of these perspectives and their dissemination. Through environmental sampling, cultivation, metagenomics, phylogenetics, and bioinformatics, our research, teaching, and outreach efforts will focus increasingly on the important awareness that all life is, put simply, one with the microbial world.

## CONFLICT OF INTEREST

The authors declare no conflicts of interest.

## Data Availability

No experimental data were generated or used in this experiment.
